# Transcriptional network analysis on brains reveals a potential regulatory role of *PPP1R3F* in autism spectrum disorders

**DOI:** 10.1186/s13104-018-3594-0

**Published:** 2018-07-17

**Authors:** Abolfazl Doostparast Torshizi, Jubao Duan, Kai Wang

**Affiliations:** 1 Raymond G. Perelman Center for Cellular and Molecular Therapeutics, Children’s Hospital of Philadelphia, Philadelphia, PA 19104 USA; 2 Center for Psychiatric Genetics, North Shore University Health System, Evanston, IL 60201 USA; 3 Department of Psychiatry and Behavioral Neurosciences, The University of Chicago, Chicago, IL 60015 USA; 4 Department of Pathology and Laboratory Medicine, Perelman School of Medicine, University of Pennsylvania, Philadelphia, PA 19104 USA

**Keywords:** Autism spectrum disorders, RNA-Seq, Next generation sequencing, Network deconvolution, Gene expression

## Abstract

**Objective:**

This study aims at identifying master regulators of transcriptional networks in autism spectrum disorders (ASDs).

**Results:**

With two sets of independent RNA-Seq data generated on cerebellum from patients with ASDs and control subjects (N = 39 and 45 for set 1, N = 24 and 38 for set 2, respectively), we carried out a network deconvolution of transcriptomic data, followed by virtual protein activity analysis. We identified PPP1R3F (Protein Phosphatase 1 Regulatory Subunit 3F) as a candidate master regulator affecting a large body of downstream genes that are associated with the disease phenotype. Pathway analysis on the identified targets of *PPP1R3F* in both datasets indicated alteration of endocytosis pathway. Despite a limited sample size, our study represents one of the first applications of network deconvolution approach to brain transcriptomic data to generate hypotheses that may be further validated by large-scale studies.

**Electronic supplementary material:**

The online version of this article (10.1186/s13104-018-3594-0) contains supplementary material, which is available to authorized users.

## Introduction

Autism spectrum disorders (ASD) comprise a set of highly inheritable neurodevelopmental conditions characterized by impairments in social communication, repetitive behaviors and restricted interests [1, 2]. ASDs are estimated to affect 1 in 68 children in the United States, and boys are 4.5 times more likely than girls to develop ASDs [3]. Several studies showed that the heritability of autistic phenotypes is estimated to be around 90% [4, 5]. The number of genes potentially implicated in ASDs is rapidly growing, mainly from large-scale genetic studies such as next generation sequencing (NGS) [6–12] and genome-wide association studies (GWAS) [13–16]. Although these studies have substantially advanced our understanding of the etiology of ASDs, the underlying molecular mechanisms remain elusive [17]. Transcriptome analysis is gaining momentum as a complementary approach to genetic association studies [17], enabling us to understand the molecular pathophysiology of ASDs.

A number of studies have evaluated whole-genome gene expression that may contribute to the onset of ASD. In a large-scale RNA-Seq effort, matched brain regions from subjects affected with ASDs and controls were utilized to identify neuronal genes strongly dysregulated in cortical regions [17]. Utilizing microarray technology, Voineagu et al. [18] demonstrated consistent differences in transcriptome organization between autistic/normal human brain tissues using gene co-expression network analysis. However, the potential molecular drivers of co-expressed modules have not been identified [18]. Despite applications of co-expression network approaches in the inference of regulatory machinery in ASD [19], state-of-the-art approaches such as network deconvolution methods are barely adopted in this area. Network deconvolution methods have been successfully used to study prostate differentiation [20] and cancers [21]. They can overcome limitations of the existing methods such as connecting genes with indirect interactions leaving their mutual causal effects aside as well as suffering from the exponentially increasing computational cost, etc. [22]. These methods can illuminate the underlying transcription circuitry of diseases and illustrate potential regulation drivers. For example, with transcriptional network deconvolution approach, we have recently provided novel insights on post-traumatic stress disorder (PTSD) [23] by identifying several genes as drivers of innate immune function. In the current study, we used ARACNe (algorithm for reconstruction of accurate cellular networks) [24] to deconvolve cellular networks. In this approach, gene–gene co-regulatory patterns are first identified using mutual information (MI), and the constructed networks are further pruned by removing indirect connections where two genes are co-regulated through one or more intermediaries. Using two of the largest transcriptomic datasets of postmortem brain tissues from ASD individuals and control subjects by Parikshak et al. [19] and Gupta et al. [17], we reconstructed the transcriptional networks followed by virtual protein activity analysis, to identify “master regulators” (MRs) that may differentially regulate the expression levels of multiple downstream genes in the cerebellum region of ASD individuals and controls.

## Main text

### Methods

Network construction and analysis tools are explained in the Additional file 1. Upon constructing the transcriptional networks, we used an algorithm called VIPER (virtual inference of protein-activity by enriched regulon analysis [21]). VIPER aims at inferring the protein activity of a MR by a systematic analysis of the expression patterns of its targets (regulons). VIPER directly integrates target mode of regulation indicating whether targets are repressed or activated given the statistical confidence in regulator–target interactions and target overlap between different regulators in order to obtain the enrichment of a protein regulon in differentially expressed genes [23]. Compared to the existing approaches such as T-profiler [25], gene set enrichment analysis (GSEA) [26], and Fisher’s exact test [27], VIPER supports seamless integration of genes with different likelihoods of representing activated, repressed or undetermined targets.

**Fig. 1:**
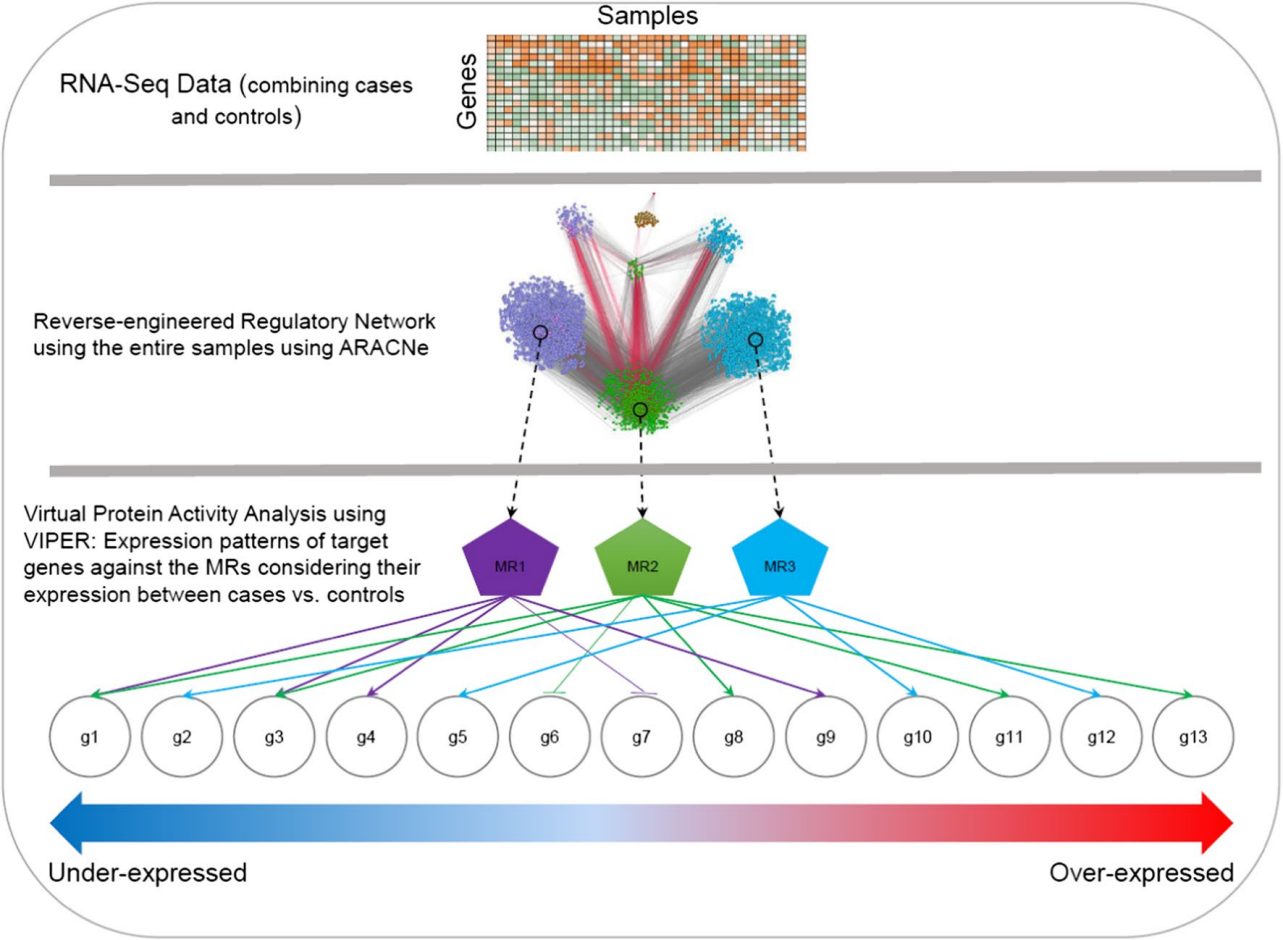
The overall process of network construction and virtual protein activity analysis to identify a master regulator

Both datasets contain multiple regions including cerebellum, which is relevant for ASDs since specific cerebellar zones can affect neocortical substrates for social interaction and cognitive functions such as language and executive functions [28–30]. Abnormalities of the cerebellum, which is believed to be involved in cognitive functions, can in part underlie autistic symptoms [31]. Several other brain regions, such as gyral surface of the anterior cingulate cortex and ventromedial prefrontal cortex [32], posterior superior temporal sulcus (pSTS) [33], amygdala, orbital frontal cortex, and fusiform gyrus [34] are also known to be ASD-relevant. We reasoned that in the same brain region, there should be highly active proteins whose expression regulates a large set of target genes and such patterns should be replicated in an independent dataset. Our preliminary finding indicates *PPP1R3F* (Protein Phosphatase 1 Regulatory Subunit 3F) as a potential master regulator (MR). The framework of the in silico experiments is illustrated in Fig. 1. Influence of dysregulation of this gene on ASD pathogenesis was then examined.

## Results and discussion

We first used the data from Parikshak et al. [19] to construct the regulatory networks. This data is part of a large RNA-Seq repository on post-mortem human brain tissue (39 cases vs. 45 controls) from cerebellum, frontal cortex, temporal cortex, prefrontal cortex, and visual cortex. During the process of network deconvolution (see Methods in Additional file 1), pairwise MI between all of the available transcripts were obtained. Next, the constructed network was trimmed to remove genetic intermediaries, resulting in potential direct connections between MRs and their targets (we used the recommended P value threshold of 10^−8^, as a measure of confidence of regulatory relationships between two genes [24]). This analysis yielded a repertoire of 672,973 interactions, 23,935 regulators, and 24,847 targets in the constructed network using the dataset from Parikshak et al. [19]. We similarly analyzed the second dataset from Gupta et al. [17], a RNA-Seq data of post-mortem brain tissues with more samples of cerebellum region than other brain regions. Using the same network construction settings on this dataset [17] containing 24 cases and 38 controls, we deconvolved a network of 297,870 interactions containing 12,040 regulators and 12,529 targets. Both constructed networks are provided in Additional files 2 and 3.

**Fig. 2:**
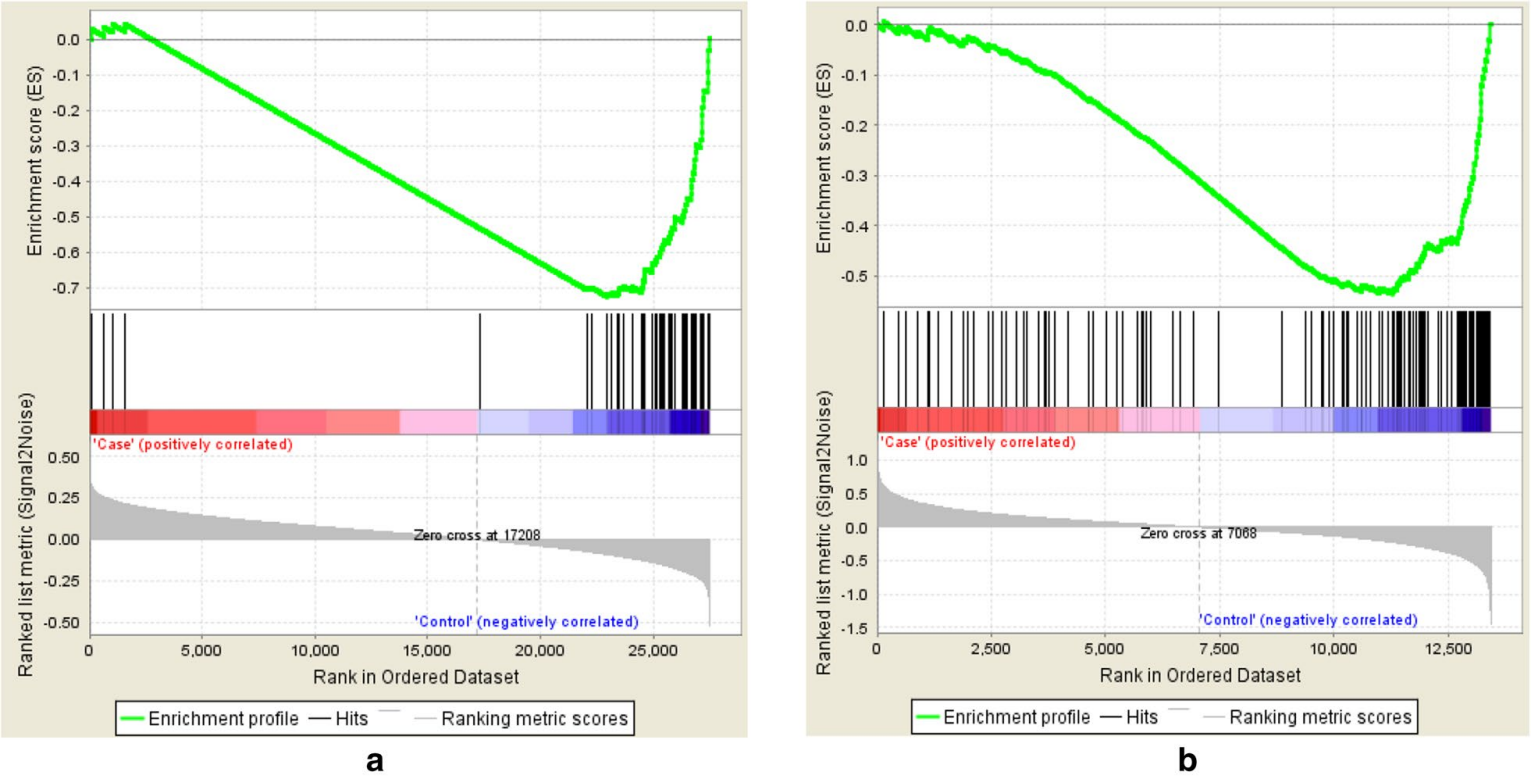
Gene set enrichment analysis (GSEA) of *PPP1R3F* targets in the constructed networks using the data by **a** Parikshak et al. [16] and **b** Gupta et al. [14]. Black bars in the both figures depict the rank of the *PPP1R3F* targets in terms of correlation with the phenotype among the entire list of genes in the both datasets

After applying VIPER, we compared the list of significant MRs at FDR ≤ 0.05. We identified *PPP1R3F* as the only MR shared between the two datasets. Given the small sample size of the data, it is possible that our analysis was underpowered and may have missed other relevant MRs in ASDs. Figure 2 illustrated how downregulation of this MR influences the expression of its regulons in the constructed networks of both data sets. *PPP1R3F* was significantly downregulated in Parikshak et al. data (FDR from one-sided *t* test: 0.029) as well as Gupta et al. data (FDR = 3.58 × 10^−4^).

*PPP1R3F* is one of the type-1 protein phosphatase (PP1) regulatory subunits. Protein phosphorylation is a key mechanism by which cells regulate signaling transduction pathways, and PPP1 family enzymes are associated with dephosphorylation of several proteins such as TGF-ß cascade [35]. *PPP1R3F* has been found to be important to neuronal activities [36]. A systematic resequencing of X-chromosome synaptic genes in a group of individuals with ASD (122 males and 20 females) has identified a rare non-synonymous variant in *PPP1R3F* that can predispose to developing ASDs [36]. This potentially damaging variant, c.733T > C, was observed in a boy with a diagnosis of asperger syndrome and was transmitted from a mother who suffered from learning disabilities and seizures [36].

Further, we examined the overlaps between *PPP1R3F* regulons and known candidate genes implicated in ASD and its related disorders (Table 1). The most significant overlap was found with SFARI gene list [37] (P = 8 × 10^−4^), followed by overlap with an intellectual disability database gene list (P = 0.072) [38]. The overlaps with other ASDs candidate gene lists also showed trends towards to being significant. These results suggest the potential relevance of the predicted *PPP1R3F* network to ASDs.

**Table 1:** The overlap between the identified *PPP1R3F* regulons from both datasets (n = 177 genes) and several candidate gene lists of ASDs and ID (intellectual disability)

Source of gene list	# Genes in the gene list	Overlap	P value	Fold enrichment	References
SFARI gene list (v 2.0)	881	17	0.0008	2.4	[37]
Intellectual disability database, University of Colorado Denver	1095	11	0.268	1.2	[41]
Intellectual disability database, University of Chicago	1969	22	0.072	1.4	[38]
Intellectual disabilities (IDS v. 1.0)	897	11	0.097	1.5	[42]
ASD de novo mutation list (v. 1.5)^a^	1248	11	0.124	1.1	[43]

P values are calculated by two-sided Fisher’s exact test

^a^We have removed de novo mutations in intergenic and intronic regions

Since *PPP1R3F* is a sex-linked gene, we accounted for differences between its expression in male and female samples with ASDs. In the Parikshak et al. data set (from Ref. [19]) there were 32 males and 7 females with ASDs while there were 39 male controls compared to 6 female controls. The gender information is not available on the Gupta et al. dataset [17]. We found no difference of *PPP1R3F* expression between male and female samples with ASDs in the Parikshak et al. dataset [19] (FDR = 0.644; two-sided *t* test), although this may be due to the small sample size. Nevertheless, to account for possible sex effects on the structure of the constructed network, we re-constructed the regulatory network using only male samples in the Parikshak et al. dataset [19] (i.e., 32 cases and 39 controls). Following the virtual protein activity analysis, we observed that *PPP1R3F* remained as a significant MR (VIPER enrichment P value = 0.0186). We note that constructing a network by using only female samples is significantly underpowered and leads to an unreliable network with a large number of false positive connections. These suggest that *PPP1R3F* likely acts independently from potential sex-based gene expression differences, and our observation of *PPP1R3F* as a MR was unlikely to be a sex-related artifact. Additionally, we conducted the same analyses on the gene expression data from prefrontal cortex, and did not find *PPP1R3F* as a significant MR (activity FDR = 0.1364). We should note that the number of samples from other brain regions were too small to be used for network analysis. Our finding thus suggests a potential role of *PPP1R3F* in developing ASDs by modulating a large body of genes in the cerebellum region.

We next conducted pathway enrichment analysis on the *PPP1R3F* regulons from both networks. We found that the gene targets are enriched for endocytosis pathway in both the Parikshak et al. dataset [19] (FDR = 5 × 10^−3^, fold enrichment = 8.26) and the Gupta et al. dataset [17] (FDR = 8 × 10^−4^, fold enrichment = 8.42). “Endocytosis” is the only significantly enriched pathway on both data sets. Combining both sets of gene targets (n = 177) (Supplementary Fig. 1 in Additional files 4 and 5) yielded a more significant enrichment of the endocytosis pathway (FDR = 4.85 × 10^−4^, fold enrichment = 8.97).

Since ASDs are commonly recognized as brain disorders, we further examined whether the identified MR is mainly expressed in the brain. We looked up *PPP1R3F* in GTEx consortium portal [39], and found that compared to other tissues, *PPP1R3F* is predominantly expressed in various brain regions such as frontal cortex and cerebellum (Supplementary Fig. 2 in Additional file 4). We also checked BrainSpan Atlas of the Developing Human Brain (http://brainspan.org) where we found that *PPP1R3F* is not expressed until 37 weeks post-conception. While remaining unexpressed in some brain regions, it is modestly expressed in 4 month postnatal stage in some brain regions including cerebellum. We further probed the expression of each of the 177 targets of *PPP1R3F* in GTEx and identified the tissues in which they are highly expressed. We found that 89 out of the 177 target genes of *PPP1R3F* are highly expressed in various brain regions compared to other tissues (P= 5.51 × 10^−5^, Fisher’s exact test; number of protein coding genes in GTEx = 20,900, number of protein coding genes highly expressed in the brain in GTEx = 7528). The enrichment of the expressed *PPP1R3F* target genes for those highly expressed in the brain supports the pathophysiological relevance of *PPP1R3F* to ASDs.

## Conclusions

In this study, we performed exploratory analysis on two small-scale RNA-Seq data sets, and used a network deconvolution algorithm to construct regulatory networks. Applying virtual protein activity analysis on both networks, we identified *PPP1R3F* as a MR of 177 targets genes. Gene set enrichment analysis on the *PPP1R3F* regulons suggested that *PPP1R3F* may exert its functional effects through regulating endocytosis, a pathway that has been previously implicated in neuropsychiatric disorders [40].

## Limitations

We acknowledge that our study is limited by the small sample size (due to the scarcity of brain tissues), and the results thus need further replications. Nonetheless, our study generates a testable hypothesis that may be validated by large-scale studies in the future. Additionally, further experimental validation of the regulatory effects of *PPP1R3F* on its downstream targets as predicted by our network analysis may provide novel insights on possible pathophysiological role of *PPP1R3F* as a MR of ASD gene network.

## Additional files

**Additional file 1.** Detailed explanation of the methods being used in this study.

**Additional file 2.** The constructed networks from the Parikshak et al. dataset [19].

**Additional file 3.** The constructed networks from the Gupta et al. dataset [17].

**Additional file 4.** Supplementary figures.

**Additional file 5.** The list of the combined set of target genes of *PPP1R3F*.

## Abbreviations

ASDs: autism spectrum disorders
NGS: next generation sequencing
GWAS: genome-wide association studies
PTSD: post-traumatic stress disorder
ARACNe: algorithm for reconstruction of accurate cellular networks
MI: mutual information
MR: master regulator
VIPER: virtual inference of protein-activity by enhanced regulon analysis
GSEA: gene set enrichment analysis
