# Supplementary material for: Transcriptional network analysis on brains reveals a potential regulatory role of PPP1R3F in autism spectrum disorders
Source: BMC Res Notes. 2018 Jul 17;11:489. doi: 10.1186/s13104-018-3594-0 (PMC6050725; doi:10.1186/s13104-018-3594-0)
Supplement: Supplementary file 1 — Additional file 1. Detailed explanation of the methods being used in this study. [file 13104_2018_3594_MOESM1_ESM.docx]

**Additional File 1**

# **Methods**

### ***RNA-Seq data***

RNA-Seq data (read counts) by Parikshak *et al* [1] was directly downloaded from PsychENCODE knowledge portal using Synapse Python client under Synapse ID syn4587609 (https://www.synapse.org/#!Synapse:syn4587609). Tissue samples for this study were acquired from the Autism Tissue Program (ATP) brain bank at the Harvard Brain and Tissue Bank and the National Institute for Child Health and Human Development (NICHD) Eunice Kennedy Shriver Brain and Tissue Bank for Developmental Disorders. The brain tissues being used to generate the RNA-Seq data include: cerebellum, frontal cortex, temporal cortex, prefrontal cortex, and visual cortex. To preserve the homogeneity of the analysis between the first dataset by Parikshak *et al* [1] and the data by Gupta *et al* [2], we initially only used the samples from cerebellum. (Later we also examined data from prefrontal cortex, to evaluate whether the candidate MR was also a significant MR in a different tissue.) The downloaded counts were z-normalized for each gene to account for the differences between the lengths of the genes given the performed sequencing depth. To do so, for each transcript across the entire samples, mean and standard deviation of the counts were obtained. Then, for each transcript across the whole samples, counts were subtracted the mean and then divided by the standard deviation. Later, the entire normalized were log2 transformed during the network construction process. Detailed procedure of tissue collection, sample preparation, data generation and processing can be found in the PsychenENCODE Knowledge Portal https://www.synapse.org/#!Synapse:syn4587609).

The second pre-processed RNA-Seq data was obtained from the study by Gupta *et al*. [2]. The data being used from their study was generated from post-mortem human brain tissues. The pre-processed data has been normalized and log-transformed and was directly used in the network construction process. The data can be accessed at <http://www.arkinglab.org/resources/>. We note that the both datasets used in this study have been normalized the same way for each brain region.

### ***Network Deconvolution***

ARACNe (Algorithm for the Reconstruction of Accurate Cellular Networks) [3], an information-theoretic algorithm for reverse engineering transcriptional networks, was used to identify candidate transcriptional regulators of the transcripts annotated to genes. First, mutual interaction between a candidate TF($x$) and its potential target ($y$) was computed by pairwise mutual information, $MI(x,y)$, using a Gaussian kernel estimator. MI was thresholded based on the null-hypothesis of statistical independence (P<0.05, Bonferroni corrected). Other key elements such as kernel width of the estimator can be set manually or automatically in the code (automated options were used in this study as recommended by the developers of the algorithm). Second, the constructed network was trimmed by removing indirect interactions by data processing inequality (DPI), a property of the MI. Based on the input parameters, the final output of ARACNe is the adjacency matrix of the constructed network that can be used for further analysis such as evaluation of protein activities. ARACNe receives the gene expression data as a text file and outputs and adjacency matrix comprising source nodes, their corresponding targets, and the MI values for each established edges.

### ***Virtual Protein Activity Analysis***

VIPER v.1.12.0 [4] was used to obtain the enrichment of regulons on gene expression signatures of the identified MR. The final outcome of the VIPER is a list of MRs based on their enrichment scores along with their respective targets. In VIPER, first, the gene expression signature is obtained by comparing two groups of samples. Student’s t-test is used to generate a quantitative measurement of difference between the groups. Next, regulon enrichment on the gene expression signature is computed using Analytic rank-based enrichment analysis (aREA). Finally, significance values (P-value and normalized enrichment score) are computed by comparing each regulon enrichment score to a null model generated by randomly and uniformly permuting the samples 1,000 times. As an internal function in VIPER, aREA tests for a global shift in the positions of each regulon genes when projected on the rank-sorted gene expression signature. Following up on the work in [5, 6], the mean of the quantile-transformed rank positions as test statistic (enrichment score) are used. The enrichment score is computed twice: (1) by a one-tail approach, based on the absolute value of the gene expression signature (i.e., genes are rank-sorted from the less invariant between groups to the most differentially expressed, regardless of the direction of change); (2) by a two-tail approach, where the positions of the genes whose expression is repressed by the regulator are inverted in the gene expression signature before computing the enrichment score. The one-tail and two-tail enrichment score estimates are integrated while weighting their contribution based on the estimated mode of regulation through a procedure we call three-tail approach. The contribution of each target gene from a given regulon to the enrichment score is also weighted based on the regulator-target gene interaction confidence. At the end, the statistical significance of the enrichment scores are estimated by comparison to a null model generated by permuting the samples uniformly at random. VIPER takes the gene expression data, phenotype information, and the constructed network. The final outcome of the VIPER is a list of MRs based on their enrichment scores along with their respective targets. VIPER software package is available on Bioconductor at http://bioconductor.org/packages/viper/.

### ***Pathway enrichment and GO analysis***

Pathway enrichment and GO analysis were conducted using WebGestalt [7]. KEGG was used as the functional database and the list of expressed genes was used as the background. Maximum (minimum) number of genes for each category was set to 2000 (5) based on the default setting. Bonferroni-Hochberg (BH) multiple test adjustment was applied to the enrichment output.

**References**

1. Parikshak, N.N., et al., *Genome-wide changes in lncRNA, splicing, and regional gene expression patterns in autism.* Nature, 2016. **540**(7633): p. 423-427.

2. Gupta, S., et al., *Transcriptome analysis reveals dysregulation of innate immune response genes and neuronal activity-dependent genes in autism.* Nat Commun, 2014. **5**: p. 5748.

3. Margolin, A.A., et al., *Reverse engineering cellular networks.* Nat Protoc, 2006. **1**(2): p. 662-71.

4. Alvarez, M.J., et al., *Functional characterization of somatic mutations in cancer using network-based inference of protein activity.* Nat Genet, 2016. **48**(8): p. 838-47.

5. Kim, S.Y. and D.J. Volsky, *PAGE: Parametric analysis of gene set enrichment.* Bmc Bioinformatics, 2005. **6**.

6. Tian, L., et al., *Discovering statistically significant pathways in expression profiling studies.* Proceedings of the National Academy of Sciences of the United States of America, 2005. **102**(38): p. 13544-13549.

7. Wang, J., et al., *WEB-based GEne SeT AnaLysis Toolkit (WebGestalt): update 2013.* Nucleic Acids Res, 2013. **41**(Web Server issue): p. W77-83.
