# Supplementary material for: Transcriptional network analysis on brains reveals a potential regulatory role of PPP1R3F in autism spectrum disorders
Source: BMC Res Notes. 2018 Jul 17;11:489. doi: 10.1186/s13104-018-3594-0 (PMC6050725; doi:10.1186/s13104-018-3594-0)
Supplement: Supplementary file 4 — Additional file 4. Supplementary figures. [file 13104_2018_3594_MOESM4_ESM.docx]

**Additional File 2**

| 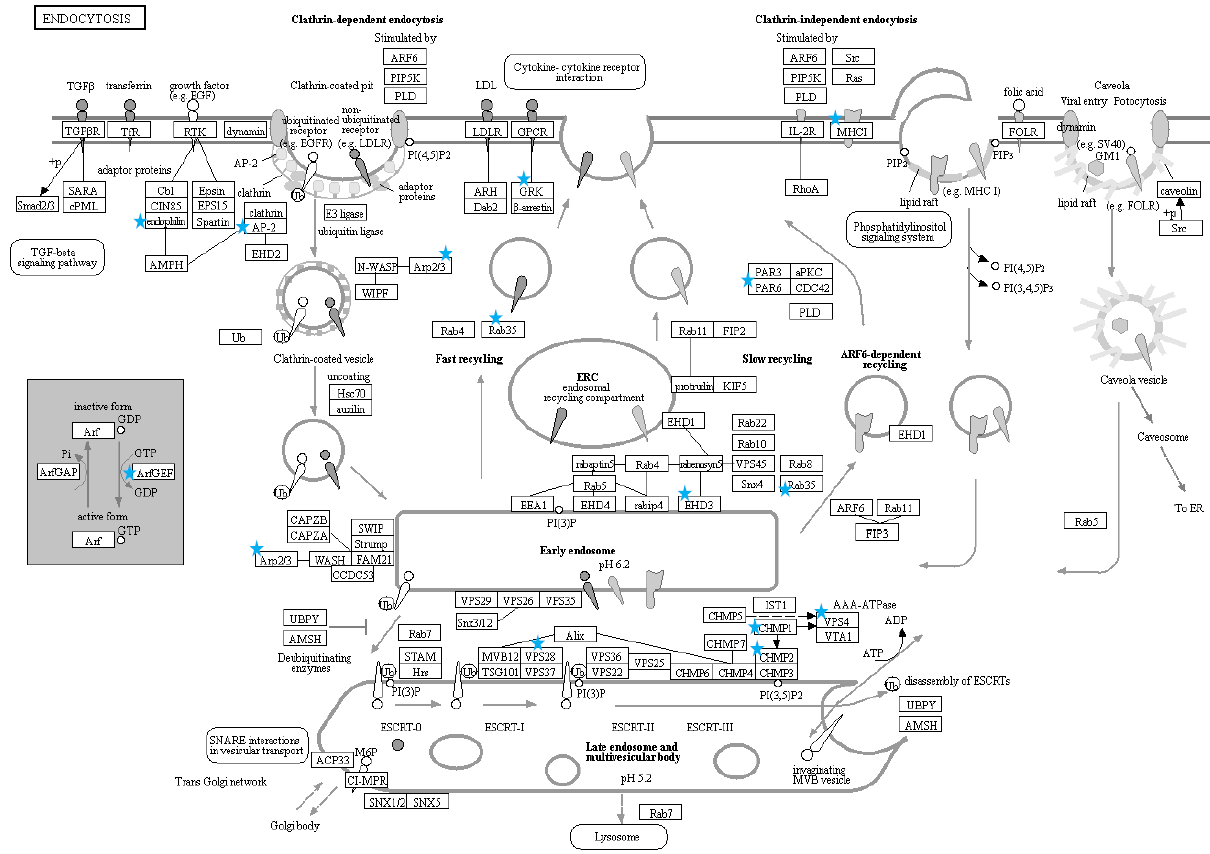 |
| --- |
| Supplementary Figure 1. Illustration of the endocytosis pathway from the KEGG pathway database (http://www.genome.jp/kegg/pathway.html). The transcriptional targets of *PPP1R3F* in both constructed networks from the data by Parikshak *et al* [16] and Gupta *et al* [14] are marked by blue stars. |


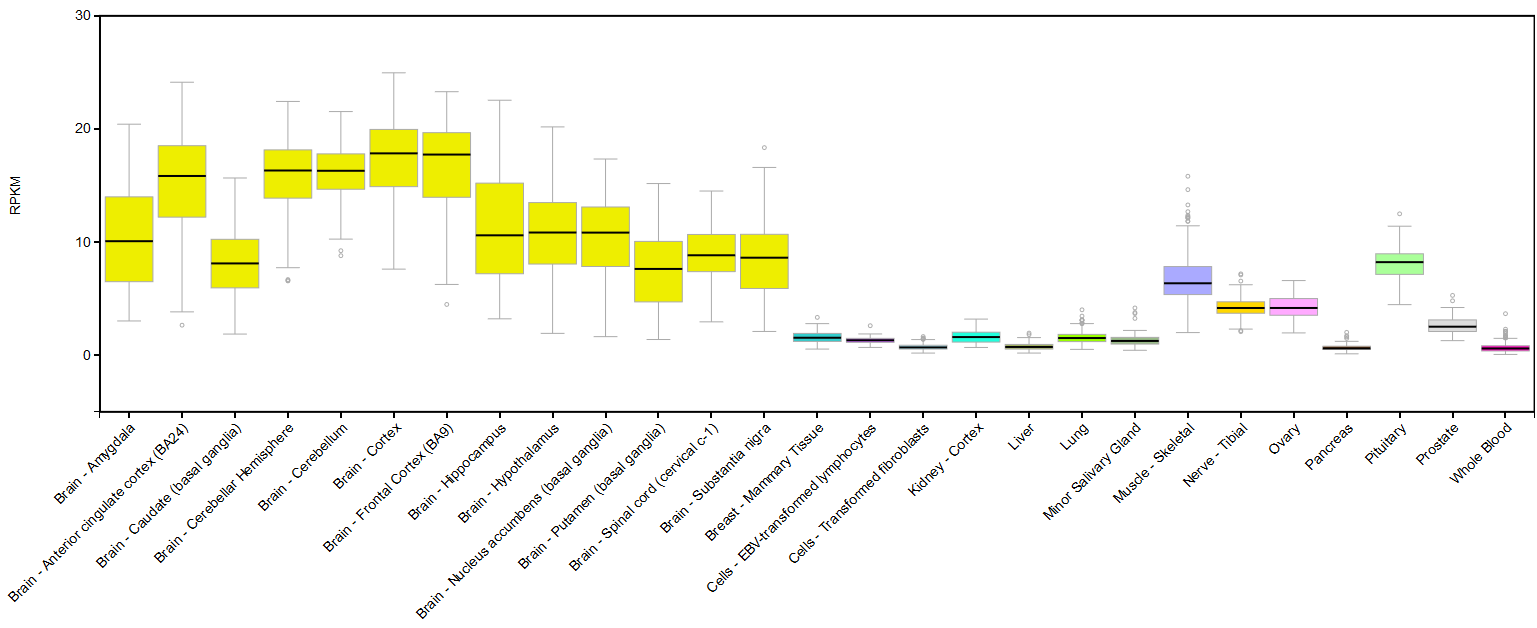


Supplementary Figure 2. *PPP1R3F* expression in various human tissues from GTEx
